# A new millipede-parasitizing horsehair worm, *Gordius chiashanus* sp. nov., at medium altitudes in Taiwan (Nematomorpha, Gordiida)

**DOI:** 10.3897/zookeys.941.49100

**Published:** 2020-06-16

**Authors:** Ming-Chung Chiu, Chin-Gi Huang, Wen-Jer Wu, Zhao-Hui Lin, Hsuan-Wien Chen, Shiuh-Feng Shiao

**Affiliations:** 1 Department of Biology, Kobe University, Kobe 657-8501, Japan Kobe University Kobe Japan; 2 Current address: Department of Biology, National Changhua University of Education, Changhua City 55007, Taiwan National Changhua University of Education Changhua Taiwan; 3 National Mosquito-Borne Diseases Control Research Center, National Health Research Institutes, Tainan City 704, Taiwan National Health Research Institutes Tainan Taiwan; 4 Department of Earth and Life Science, University of Taipei, Taipei 100, Taiwan University of Taipei Taipei Taiwan; 5 Department of Entomology, National Taiwan University, Taipei 106, Taiwan National Taiwan University Taipei Taiwan; 6 Department of Biological Resources, National Chiayi University, Chiayi 300, Taiwan National Chiayi University Chiayi Taiwan

**Keywords:** definitive host, immature stage, parasitic life cycle, terrestrial adaptation

## Abstract

*Gordius chiashanus***sp. nov.**, a newly described horsehair worm that parasitizes the *Spirobolus* millipede, is one of the three described horsehair worm species in Taiwan. It is morphologically similar to *G. helveticus* Schmidt-Rhaesa, 2010 because of the progressively broadening distribution of bristles concentrated on the male tail lobes, but it is distinguishable from *G. helveticus* because of the stout bristles on the mid-body. In addition, a vertical white stripe on the anterior ventral side and areoles on the inside wall of the cloacal opening are rarely mentioned in other *Gordius* species. Free-living adults emerged and mated on wet soil under the forest canopy in the winter (late November to early February) at medium altitudes (1100–1700 m). Mucus-like structure covering on the body surface, which creates a rainbow-like reflection, might endow the worm with high tolerance to dehydration. Although *Gordius chiashanus***sp. nov.** seems to be more adaptive to the terrestrial environment than other horsehair worm species, cysts putatively identified as belonging to this hairworm species found in the aquatic paratenic host, *Ephemera orientalis* McLachlan, 1875, suggest the life cycle of *Gordius chiashanus***sp. nov.** could involve water and land. The free-living adults emerged from the definitive hosts might reproduce in the terrestrial environment or enter an aquatic habitat by moving or being washed away by heavy rain instead of manipulating the behavior of their terrestrial definitive hosts.

## Introduction

In addition to the two previously described species of horsehair worm (Chiu et al. 2011, 2017), *Gordius chiashanus* sp. nov. is the third described species in Taiwan, and one among 90 valid *Gordius* species reported worldwide (Schmidt-Rhaesa 2010, 2014). *Gordius* horsehair worms are characterized by a cuticular fold, known as postcloacal crescent, on the male tail (Schmidt-Rhaesa 2002). *Gordius* forms a monophyletic group (Gordiidae) with the genus *Acutogordius*, which bears the same characteristics; however, the phylogenetic relationship between these two genera is controversial (Schmidt-Rhaesa 2002). Although *Gordius* is the second most diverse genus, identification of species in the genus *Gordius* is difficult because of the lack of diagnostic characters and our limited understanding of its morphological variables (Schmidt-Rhaesa 2001, 2010). Phylogenetic comparison using DNA sequences with morphological descriptions has become increasingly crucial in detecting the cryptic species (Hanelt et al. 2015; Tobias et al. 2017).

The definitive hosts of *Gordius* cover a wide range of arthropod taxa. Although many host records might be questionable because the genus *Gordius* (*G. aquaticus* Linnaeus, 1758) had been used to represent the entire members of horsehair worms, *Gordius* species might parasitize several insect orders, Chilopoda, Diplopoda, and Araneae as their definitive hosts (Schmidt-Rhaesa 2012; Bolek et al. 2015). The *Gordius* life cycle is highly correlated with the definitive hosts. The freshwater horsehair worm typically exhibits a life cycle that involves aquatic and terrestrial environments; its life cycle comprises a reproduction and paratenic aquatic host phase and a terrestrial definitive host phase (Hanelt et al. 2005). The aforementioned complex life cycle has been reported in multiple *Gordius* species (e.g., *G. robustus* Leidy, 1851 and *G. difficilis* Smith, 1994) (Thorne 1940; Bolek and Coggins 2002); however, it has not been reported in some species that parasitize aquatic definitive hosts (e.g., *G. villoti* Rosa, 1882 and *G. albopunctatus* Müller, 1926) (Valvassori et al. 1988; Schmidt-Rhaesa and Kristensen 2006) or in species that reproduce in terrestrial environments (*G. terrestris*Anaya et al., 2019) (Anaya et al. 2019).

Free-living adults of *Gordius chiashanus* sp. nov. are frequently found in foggy forests situated at altitudes of 1100–1700 m in Taiwan. Their taxonomic status was first examined in the present study by using a description of morphology and phylogenetic comparison of partial mitochondrial DNA cytochrome oxidase subunit I (mtDNA-COI) genes. The definitive host was determined using worms with high sequence similarity collected from the round-backed millipede, *Spirobolus* sp. nov. (Hsu and Chang, unpublished). Egg strings and larvae were obtained by allowing a field collected adult free-living female worm to oviposit egg string in the laboratory. The cysts which morphologically similar to the laboratory-reared larvae were collected from the field-collected mayfly naiad, *Ephemera orientalis* McLachlan, 1875. Based on our field observations on adult free-living worms, cysts and their hosts, along with our laboratory observations of non-adult stages for this gordiid species, we suggest the possible life history of *Gordius chiashanus* sp. nov.

## Materials and methods

### Collection and preservation of horsehair worms

Horsehair worm samples were identified visually and collected from the ground. In total, 21 free-living adults (17 male and 4 female adults) were collected for morphological examination and DNA sequencing (detailed information provided in Table 1). All the living worms were killed by treatment with hot water (> 80 °C), fixed in a solution containing 75% alcohol with their hosts for a few days, and preserved in a solution of 95% alcohol. One mated female adult collected from Fenqihu, Zhuqi township, Chiayi county, Taiwan (23°30'12.70"N, 120°41'36.00"E) was placed in 800 mL of aerated tap water in the laboratory and maintained at 15 °C until it oviposited egg strings. The eggs were maintained in aerated water for 49 days until they hatched. One dead worm from a dead round-backed millipede (collected at 17-III-2019) and five immature worms from three of 50 round-backed millipedes (collected at 23-VII-2018 and 28-VII-2018) were collected to confirm the definitive host (detailed information provided in Table 1). All the hosts were preserved at –20 °C until dissection. The infected host and the harbored worms were preserved in a 95% alcohol solution for sequencing. Five cysts photographed from four mayfly naiads of *E. orientalis* collected from Lugu township, Nantou county, Taiwan (23°40'46.00"N, 120°47'18.50"E), where the free-living adult has ever been found in the upstream of less than 1 km, were putatively identified as belonging to this horsehair worm species. All the samples were preserved in a solution of 75% alcohol for morphological examination.

### Morphological examination

**Free-living adults.** Fragments (approximately 0.5 cm in length) of the anterior end, mid-body, and posterior end of the preserved samples were examined and photographed using a stereomicroscope (Leica S8 APO, Leica, Wetzlar, Germany), dehydrated using a series of ethanol and acetone solutions (95% and 100% ethanol (twice) and ethanol/acetone mixtures of 2:1, 1:1, 1:2, and 0:1), dried to the critical point, coated by being sputtered with gold, and examined using a scanning electronic microscope (SEM) (JEOL JSM-5600, Tokyo, Japan) at magnifications ranging from 100× to 15,000×.

**Eggs and larvae.** Eggs and newly hatched larvae (living or treated with hot water (> 80 °C)) were examined and photographed on microslides by using a compound microscope (Olympus BH-2, PM-10AD, Olympus, Tokyo, Japan) at magnifications of 200× and 400×. The eggs examined using the SEM were first fixed using a solution of 75% alcohol, dehydrated, dried to the critical point, and coated with gold sputter. The eggs and larvae were examined at a magnification of 500×. ImageJ 1.47 was used for all morphological measurements (Abràmoff et al. 2004), and spatial calibration was conducted according to the scale included in each picture. The terminology for larval stages used in this study primarily followed that of Schmidt-Rhaesa (2014) and Szmygiel et al. (2014).

**Cysts in the paratenic host.** The mayflies preserved in 75% alcohol were first treated with Nesbitt’s fluid for 15–20 min at 40 °C and a 0.1% KOH solution for 5 min at 40 °C to ensure that the cuticle and muscles had become transparent (Walter and Krantz 2009; Chiu et al. 2016). One of the cysts was further treated with a 5% KOH solution for 6 h at room temperature to release the folded larva inside the cyst wall. The cysts were examined and photographed on microslides by using the compound microscope at 200× magnification.

### Phylogenetic analysis

Genomic DNA from a 1-cm mid-body section of each worm was extracted using an ALS Tissue Genomic DNA Extraction Kit (Pharmigene, Kaohsiung, Taiwan). The partial cytochrome c oxidase subunit I (COI) sequence was amplified using universal primers (LCO1490 and HC02198) (Folmer et al. 1994) or a newly designed primer set (GoCOiF-1: TTAGGAACTGCTTTAAG, GoCOiR-1: ATAGGGTCAAAGAAGGAGG). PCR for both primer sets was initiated at 95 °C for 5 min, and amplification was conducted for 35 cycles of 95 °C for 1 min, 50 °C for 1 min, and 73 °C for 1 min, with a final extension at 73 °C for 5 min.

In addition to sequencing three free-living adult worms and six immature worms recovered from millipede hosts (242–457 high-quality base pairs), we obtained high-quality CO1 sequences (>500 base pairs) from 18 adult free-living individuals to be used in our phylogenetic analysis and estimates of intraspecific genetic distances. Pairwise distance matrices of COI sequence data were calculated using the Kimura 2-parameter model. A phylogenic tree was reconstructed using the maximum likelihood method by using the General Time Reversible model with the addition of invariant sites and a gamma distribution of rates across sites. For phylogenic analysis, the COI sequences were first aligned using CLUSTALX 2.0.10 (Thompson et al. 1997). A total of 422 base pairs shared by all the examined sequences, including for our 18 samples, *Gordius*/*Acutogordius* spp. (as reported by Sato et al. (2012), Hanelt et al. (2015), Chiu et al. (2017), and Tobias et al. (2017)) and *Chordodes formosanus* Chiu, 2011, *Euchordodes nigromaculatus* Poinar, 1991, and *Parachordodes diblastus* (Örley, 1881) (as reported by Chiu et al. (2011) and Tobias et al. (2017)), were analyzed using MEGA 7 (Kumar et al. 2016) (see detailed information in Table 2). One sequence of an undetermined nematomorph (MF983649) was also included because it exhibited high similarity to *Acutogordius*. The bootstrap method (with 1000 replicates) was used to estimate branch support of the phylogenic tree.

### Seasonal occurrence of free-living adults

Seasonal occurrence of free-living adults was estimated by counting (and removing) free-living adults (living or dead) on the ground in Dinghu, Alishan township, Chiayi county, Taiwan (23°29'29.10"N, 120°43'19.00"E) between October 2017 and May 2018.

## Results

### Gordius chiashanus

Taxon classificationAnimaliaGordeaGordiidae

Chiu sp. nov.

22146B0F-BDE4-5A45-8C99-DB1C29B01FFE

http://zoobank.org/E904851F-6F48-423D-9AC2-5A7BB595FA7B

#### Type locality.

Dinghu (23°29'29.10"N, 120°43'19.00"E), Alishan township, Chiayi county, Taiwan (holotype). Paratypes were collected from Dasyueshan (Heping district, Taichung city), Xitou (Lugu township, Nantou county), Shihjhuo, Fenqihu (Zhuqi township, Chiayi county), Dinghu (Alishan township, Chiayi county), and Hongshi forest road (Haituan township, Taitung county). Table 1 presents detailed information of the locality.

#### Type material.

Partial bodies of the holotype and allotype were deposited at the National Museum of Natural Science, Taichung, Taiwan. Paratypes were deposited at the National Museum of Natural Science, Taichung, Taiwan and Lake Biwa Museum, Shiga, Japan (Table 1).

**Table 1.** *Gordius chiashanus* sp. nov. specimen information.

Collection date	GenBank no.	Locality	Longitude and latitude	Collector	Depository	Sex	Status	Length (mm)
20-XI-2017	MN784831 ^1^	Dasyueshan (Heping, Taichung, Taiwan)	24°14'47.90"N, 120°56'06.80"E	Ta-Chih Chen	NMNS	M	Free-living adult	430
26-XI-2008	MN784832	Hongshi trail (Haituan, Taitung, Taiwan)	23°04'14.50"N, 121°07'58.30"E	Po-Yen Chen	NMNS	M	Free-living adult	744
22-I-2008	MN784841	Shihjhuo (Zhuqi, Chiayi, Taiwan)	23°29'01.70"N, 120°42'05.90"E	Yu-Hsuan Tsai	NMNS	M	Free-living adult	860
9-II-2007	MN784833	Shihjhuo (Zhuqi, Chiayi, Taiwan)	23°29'01.70"N, 120°42'05.90"E	Yu-Hsuan Tsai	NMNS	F	Free-living adult	707
8-XII-2017	MN784819	Dinghu (Alishan, Chiayi, Taiwan)	23°29'29.10"N, 120°43'19.00"E	Ming-Chung Chiu	LBM	M	Free-living adult	771
8-XII-2017	MN784820	Dinghu (Alishan, Chiayi, Taiwan)	23°29'29.10"N, 120°43'19.00"E	Ming-Chung Chiu	NMNS	M	Free-living adult	734
8-XII-2017	MN784821	Dinghu (Alishan, Chiayi, Taiwan)	23°29'29.10"N, 120°43'19.00"E	Ming-Chung Chiu	NMNS	M	Free-living adult	726
17-XII-2013	MN784822	Fenqihu (Zhuqi, Chiayi, Taiwan)	23°30'12.70"N, 120°41'36.00"E	Hua-Te Fang	LBM	M	Free-living adult	803
17-XII-2013	MN784823	Fenqihu (Zhuqi, Chiayi, Taiwan)	23°30'12.70"N, 120°41'36.00"E	Hua-Te Fang	LBM	M	Free-living adult	756
17-XII-2013	MN784824	Fenqihu (Zhuqi, Chiayi, Taiwan)	23°30'12.70"N, 120°41'36.00"E	Hua-Te Fang	NMNS	M	Free-living adult	594
17-XII-2013	MN784825	Fenqihu (Zhuqi, Chiayi, Taiwan)	23°30'12.70"N, 120°41'36.00"E	Hua-Te Fang	NMNS	M	Free-living adult	383
17-XII-2013	MN784826	Fenqihu (Zhuqi, Chiayi, Taiwan)	23°30'12.70"N, 120°41'36.00"E	Hua-Te Fang	NMNS	M	Free-living adult	676
17-XII-2013	MN784827	Fenqihu (Zhuqi, Chiayi, Taiwan)	23°30'12.70"N, 120°41'36.00"E	Hua-Te Fang	NMNS	M	Free-living adult	474
18-XII-2017	MN784828	Fenqihu (Zhuqi, Chiayi, Taiwan)	23°30'12.70"N, 120°41'36.00"E	Ming-Chung Chiu	NMNS	M	Free-living adult	749
18-XII-2017	MN784829	Fenqihu (Zhuqi, Chiayi, Taiwan)	23°30'12.70"N, 120°41'36.00"E	Ming-Chung Chiu	NMNS	F	Free-living adult	666
18-XII-2017	MN784830	Fenqihu (Zhuqi, Chiayi, Taiwan)	23°30'12.70"N, 120°41'36.00"E	Ming-Chung Chiu	NMNS	F	Free-living adult	717
18-XII-2016	MN784816	Xitou (Lugu, Nantou, Taiwan)	23°40'21.30"N, 120°47'27.50"E	Ming-Chung Chiu	LBM	M	Free-living adult	498
18-XII-2016	MN784817	Xitou (Lugu, Nantou, Taiwan)	23°40'21.30"N, 120°47'27.50"E	Ming-Chung Chiu	NMNS	M	Free-living adult	403
18-XII-2016	MN784818	Xitou (Lugu, Nantou, Taiwan)	23°40'21.30"N, 120°47'27.50"E	Ming-Chung Chiu	LBM	F	Free-living adult	549
9-II-2008	MN784842	Xitou (Lugu, Nantou, Taiwan)	23°40'21.30"N, 120°47'27.50"E	Ming-Chung Chiu	NMNS	M	Free-living adult	572
10-XII-2011	MN784840	Xitou (Lugu, Nantou, Taiwan)	23°40'21.30"N, 120°47'27.50"E	Ming-Chung Chiu	NMNS	M	Free-living adult	502
17-III-2019	MN784839	Xitou (Lugu, Nantou, Taiwan)	23°40'21.30"N, 120°47'27.50"E	Zhao-Hui Lin	NMNS	-	Dead worm in host	-
23-VII-2018	MN784834	Shihjhuo (Zhuqi, Chiayi, Taiwan)	23°29'01.70"N, 120°42'05.90"E	Yu-Wei Li	NMNS	-	Immature worm	660
28-VII-2018	MN784835	Shihjhuo (Zhuqi, Chiayi, Taiwan)	23°28'22.60"N, 120°41'42.80"E	Yu-Wei Li	NMNS	-	Immature worm	894
28-VII-2018	MN784836	Shihjhuo (Zhuqi, Chiayi, Taiwan)	23°28'22.60"N, 120°41'42.80"E	Yu-Wei Li	NMNS	-	Immature worm	420
28-VII-2018	MN784837	Shihjhuo (Zhuqi, Chiayi, Taiwan)	23°28'22.60"N, 120°41'42.80"E	Yu-Wei Li	NMNS	-	Immature worm	442
28-VII-2018	MN784838	Shihjhuo (Zhuqi, Chiayi, Taiwan)	23°28'22.60"N, 120°41'42.80"E	Yu-Wei Li	NMNS	-	Immature worm	426

LBM: Lake Biwa Museum; NMNS: National Museum of Natural Science. ^1^ Holotype.

#### Type hosts.

*Spirobolus* sp. nov. (Hsu and Chang, unpublished) (Diplopoda: Spirobolidae) (Fig. 5E, F)

#### Etymology.

The specific name is the combination of *chia*, referring to the place (Chiayi county) where the first sample was found, and *shan*, referring to the Chinese word for “mountains.” The word *chia* is also in memory of our friend, Chia-Chih Lin, who died in an accident in a field experiment.

#### Description.

**Figure 1.**
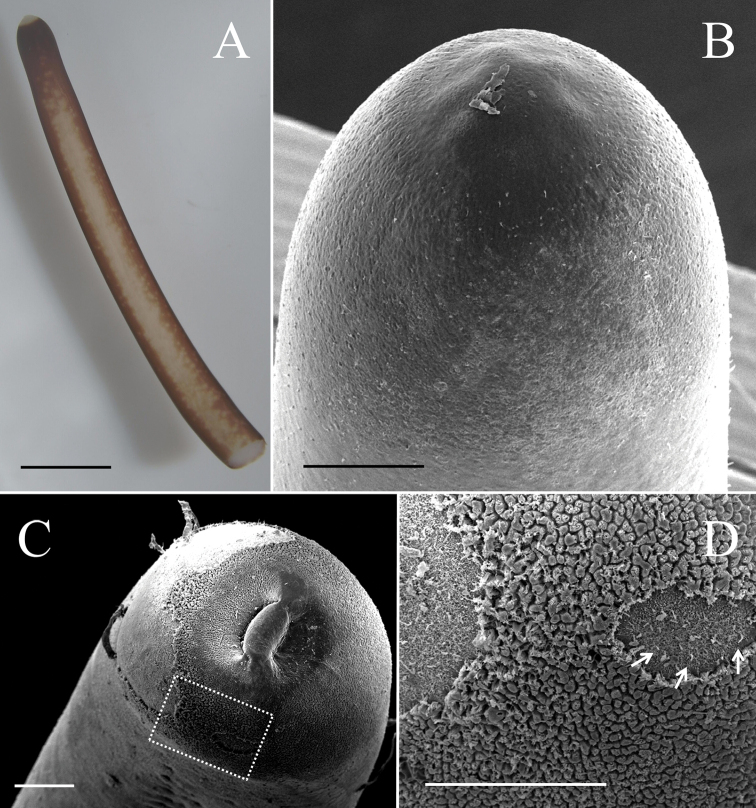
Anterior end of male *Gordius chiashanus* sp. nov. **A** stereomicroscopic image of the ventral side of the anterior end showing a white cap, dark-brown collar, and vertical white stripe on the ventral side **B, C**SEM images of the anterior end surface that is (**B**) smooth with scattered short bristles and (**C**) wrinkled **D** close-up view of the dotted square in C showing the short bristles (arrows) covered by a wrinkled structure. Scale bars: 2 mm (**A**), 200 μm (**B–D**).

**Figure 2.**
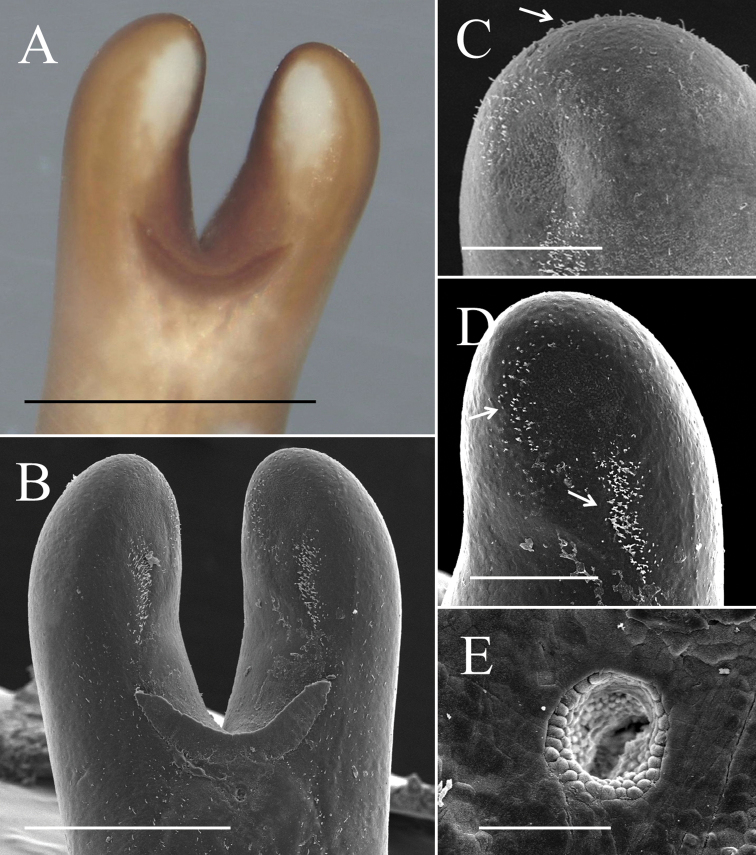
Posterior end of male *Gordius chiashanus* sp. nov. **A** stereomicroscopic image of the posterior end **B–D**SEM images of (**B**) overview of the posterior end with bristles concentrated on the (**C**) lobe tips (arrow), and (**D**) inner side of the lobe tips and the formation of a bristle field on each tail lobe posterior to the tips of the postcloacal crescent (arrows) **E** cloacal opening with areoles on the inside wall. Scale bars: 1 mm (**A**), 500 μm (**B**), 200 μm (**C–D**), 50 μm (**E**).

**Figure 3.**
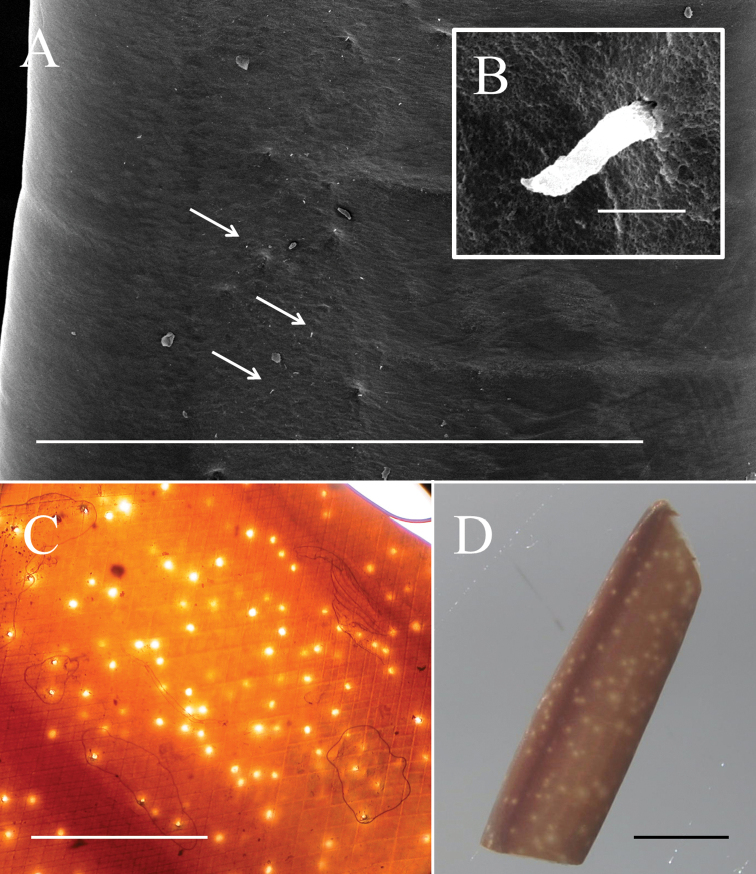
Mid-body of male *Gordius chiashanus* sp. nov. **A, B**SEM images of (**A**) cuticle in the mid-body with scattered short bristles (arrows) and (**B**) close-up view of a short bristle **C, D** white spots and dorsal and ventral dark pigmented line examined using (**C**) a compound microscope and (**D**) a stereomicroscope. Scale bars: 1 mm (**A, C, D**), 5 μm (**B**).

**Male adults (*N* = 11) (Figs 1–3, 5).** Body length 627.94 ± 154.75 (383–860) mm, width (widest, after dehydration) 1.30 ± 0.31 (0.81–2.06) mm, light to dark brown, smooth, and covered with mucus-like structure (viscous liquid on live worms with rainbow-like reflection (Fig. 5C, Suppl. material 1: Video S1), and created haze that surrounded the body surface in hot water (Fig. 5A).

Anterior end columnar and spherical; anterior tip white (white cap) with a dark -brown collar and a vertical white stripe on the ventral side (Fig. 1A). Under SEM, surface of anterior end appeared smooth (Fig. 1B) or wrinkled (Fig. 1C) on the tip of one sample; scattered short bristles (11.24 ± 6.57 (4.92–22.24) µm in length) scattered except on tip in most samples (Fig. 1B, D).

Cuticle in mid-body ornamented with a dorsal and a ventral dark pigment line; white spots scattered across entire body surface (Figs 3C, D, 5A). Under SEM, cuticle surface appeared smooth (Fig. 3A) with a few scattered short or cone-like bristles (6.75 ± 2.37 (2.31–10.34) µm in length) (Fig. 3A, B).

Posterior end divided into two tail lobes (Fig. 2A, B), each lobe 855.24 ± 100.89 (658.39–994.88) µm long and 458.55 ± 76.52 (365.95–643.00) µm wide with length-to-width ratio of 1.89 ± 0.26 (1.49–2.42). Inner side of lobe tips white (Fig. 2A). Under SEM, inner side of tail lobes concave in some samples; cuticle surface smooth, but one sample exhibited flat areoles on inner side of lobe tips; short bristles scattered across the surface and concentrated in most samples on lobe tips (Fig. 2C) and on inner side of lobe tips forming a bristle field (322.67 ± 99.34 (187.60–412.75) µm long and 71.82 ± 35.49 (44.81–114.54) µm wide) on each of tail lobe posterior to tips of postcloacal crescent (Fig. 2D). Postcloacal crescent (Fig. 2A, B) 718.61 ± 118.77 (536.14–984.34) µm long and 86.7 ±15.62 (54.73–118.65) µm wide and located on ventral side near base of tail lobes. Crescent generally semicircular or slightly angled, but a few samples exhibited a straightened form of crescent. Branches of postcloacal crescent usually ended at tail lobes. Cloacal opening circular (40.5 ± 21.87 (27.41–56.14) µm) and anterior to postcloacal crescent (Fig. 2A, B). Wall inside cloacal opening exhibited areoles (Fig. 2E); no circumcloacal spine or bristles observed in region next to cloacal opening.

**Figure 4.**
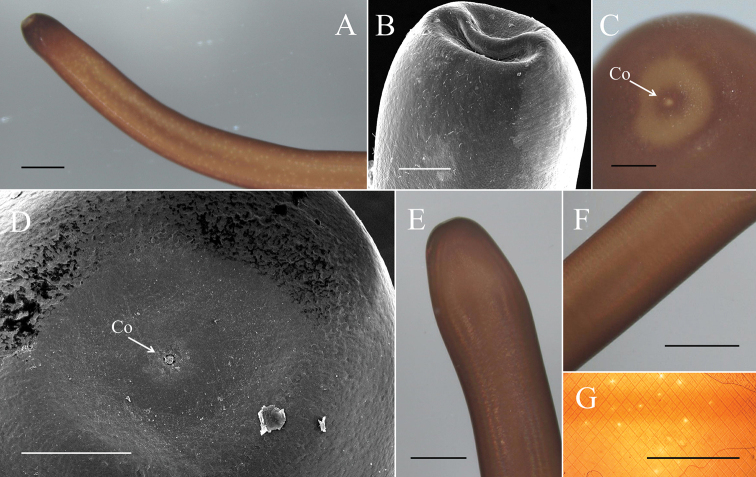
Female *Gordius chiashanus* sp. nov. **A, B** anterior end examined using a (**A**) stereomicroscope and (**B**) SEM**C–E** posterior end with the terminal view examined using a (**C**) stereomicroscope and (**D**) SEM, and the (**E**) lateral view examined using a stereomicroscope **F, G** mid-body examined using a (**F**) stereomicroscope and (**G**) compound microscope. Co, cloacal opening. Scale bars: 1 mm (**A, F, G**), 200 μm (**B–D**).

**Figure 5.**
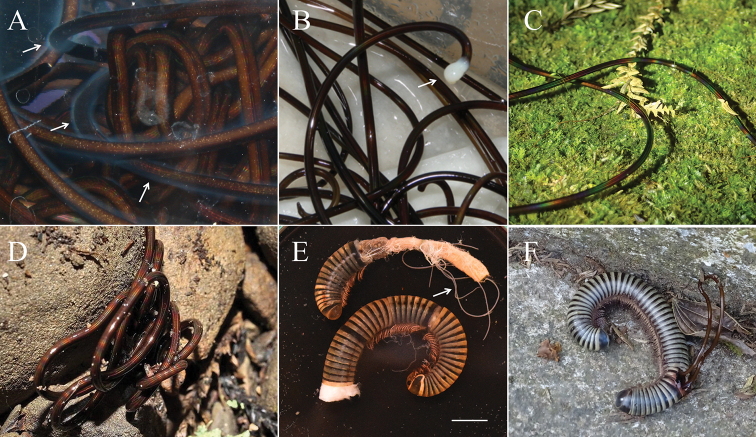
Field observation of *Gordius chiashanus* sp. nov. **A** hazy appearance (arrows) surrounding the body surface in hot water **B** spermatophore (arrow) on a female collected on the surface of the soil **C** rainbow-like reflection on the body surface **D** free-living adult collected in wet soil **E, F** infected host, *Spirobolus* sp. nov. (Hsu and Chang, unpublished), harboring (**E**) three immature worms (arrow) and (**F**) an adult worm. Photographs courtesy of (**D**) Fang, Hua-Te and (**F**) Hung, Ming-Chin. Scale bars: 1 cm (**E**).

**Female adults (*N* = 4) (Figs 4, 5).** Body length 659.75 ± 77.06 (549–717) mm, width (widest, after dehydration) 1.54 ± 0.54 (1.00–2.03) mm, light to dark brown, smooth, and covered with mucus-like structure. White spots scattered on surface but relatively less obvious than those of male adults (Fig. 4F, G). Anterior end columnar and spherical. Anterior tip white (white cap) with a dark-brown collar and exhibited a vertical white stripe on the ventral side (Fig. 4A). Under SEM, surface of anterior end smooth and exhibited scattered short bristles (16.75 ± 4.60 (13.39–23.56) µm in length) except at tip (Fig. 4B). Cuticle in mid-body ornamented with a dorsal and a ventral dark pigment line (Fig. 4G). Under SEM, cuticle surface smooth with a few short or cone-like bristles (7.24 ± 2.01 (4.94–9.99) µm in length) scattered. Posterior end columnar and rounded at tip (Fig. 4E) and did not exhibit scattered bristles (Fig. 4D). Cloacal opening on terminal end (Fig. 4C, D) circular and 36.56 ± 23.23 (24.68–48.45) µm in diameter.

**Figure 6.**
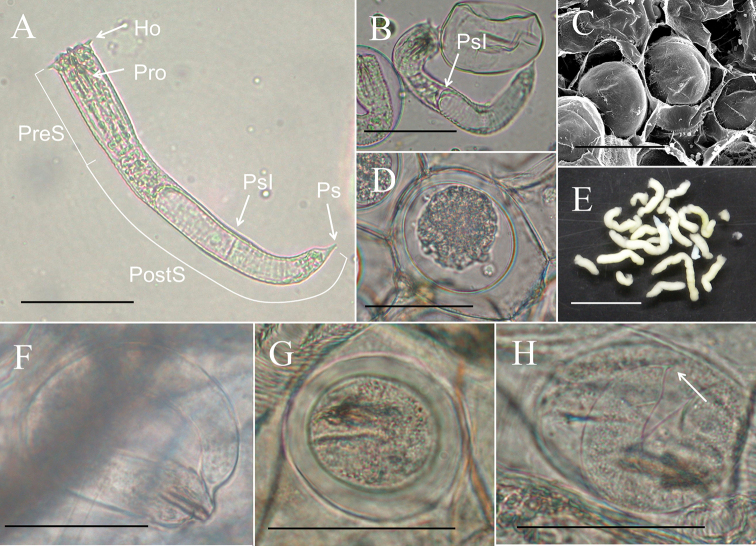
Immature stages of *Gordius chiashanus* sp. nov. **A, B** free-living larva (**A**) treated with hot water and a living larva showing the depression in the anterior end of the pseudointestine (arrow) **C, D** eggs with the inner membrane examined using an (**C**) SEM and (**D**) compound microscope **E** egg strings **F–H** cysts in the paratenic host with (**F**) a unfolded larva and (**G**) a folded larva, showing (**H**) a single posterior spine (arrow) after treatment with a 5% KOH solution. Abbreviations: Ho, hooklet; PostS, postseptum; PreS, preseptum; Pro, proboscis; PsI, pseudointestine. Scale bars: 50 µm (**A–D, F–H**), 1 mm (**E**).

**Eggs (*N* = 12) (Fig. 6C–E).** Egg strings (Fig. 6E) 7.41 ± 3.46 (3.78–13.70) mm in length and 1.13 ± 0.12 (0.86–1.25) mm in width; white or light yellow in color, deposited in water as short pieces not adhering to substrate. Eggs round, 54.16 ± 242 2.89 (49.88–58.61) µm in diameter. Developing embryo surrounded by an inner membrane (Fig. 6C, D) separated by a distinct space from outer egg shell 14.35 ± 1.41 (12.43–17.33) µm).

**Living larvae (*N* = 10) (Fig. 6B).** Eggs developed for approximately 49 days. Hatched larvae remained near egg strings or moved inside eggshells. Under light microscopy, living larvae appeared cylindrical with a single posterior spine. Preseptum length 32.33 ± 4.53 (27.06–40.04) µm, and the width 18.04 ± 0.86 (16.70–19.12) µm. Postseptum length 83.05 ± 8.31 (66.50–92.66) µm, width 15.05 ± 0.73 (14.21–16.10) µm; proboscis length 14.94 ± 1.99 (12.35–18.48) µm, width 4.11 ± 0.85 (2.77–5.34) µm; pseudointestine length 60.60 ± 5.40 (54.99–70.12) µm, width 11.66 ± 1.42 (8.84–13.56) µm, unequally subdivided, elongated oval with a depression in anterior end (Fig. 6B).

**Larvae treated with hot water (*N* = 2) (Fig. 6A).** Larvae treated with hot water similar in morphology but larger than living larvae. Preseptum length 44.57 ± 0.13 (44.48–44.66) µm, width 17.96 ± 0.16 (17.85–18.08) µm. Postseptum length 118.23 ± 1.91 (116.88–119.58) µm, width 15.36 ± 0.68 (14.88–15.84) µm. Proboscis length 12.63 ± 1.18 (11.80–13.47) µm, width 3.26 ± 0.05 (3.23–3.30) µm; pseudointestine length 77.99 ± 5.22 (74.30–81.68) µm, width 13.99 ± 0.81 (13.41–14.56) µm (Fig. 6A).

**Field-collected cysts (*N* = 5) (Fig. 6F–H) .** Larvae in cysts unfolded (*N* = 4) (Fig. 6F) or exhibited a postseptum folded twice (*N* = 1) (Fig. 6G, H). Unfolded larvae morphologically similar to larvae but larger in size; preseptum length was 60.18 ± 6.72 (50.40–65.18) µm, width 20.87 ± 0.52 (20.28–21.33) µm; postseptum length 127.33 ± 20.05 (105.10–146.05) µm, width 19.82 ± 2.27 (17.61–22.91) µm; proboscis length 15.46 ± 1.67 (13.84–17.56) µm, width 4.10 ± 0.68 (3.09–4.52) µm; pseudointestine not visible (Fig. 6F). Folded larva (length 34.97 µm, width 30.47 µm) fold twice and surrounded by a clear cyst wall, 47.86 µm in total length and 42.40 µm in total width; proboscis length 15.57 µm, width 5.09 µm (Fig. 6G); a single posterior spine visible after treatment with a solution of 5% KOH (Fig. 6H).

**Table 2.** List of COI sequences obtained from GenBank for phylogenetic analyses in this study.

Accession number	Species/clade	Reference
*Gordius*/*Acutogordius*		
KM382317	G. cf. robustus (Clade 8)	Hanelt et al. 2015
KM382316	‘’	Hanelt et al. 2015
KM382315	‘’	Hanelt et al. 2015
KM382314	‘’	Hanelt et al. 2015
KM382313	‘’	Hanelt et al. 2015
KM382312	‘’	Hanelt et al. 2015
KM382311	‘’	Hanelt et al. 2015
KM382310	*G. terrestris*	Hanelt et al. 2015, Anaya et al. 2019
KM382309	‘’	Hanelt et al. 2015, Anaya et al. 2019
KM382308	‘’	Hanelt et al. 2015, Anaya et al. 2019
KM382307	‘’	Hanelt et al. 2015, Anaya et al. 2019
KM382306	G. cf. robustus (Clade 6)	Hanelt et al. 2015
KM382305	‘’	Hanelt et al. 2015
KM382304	‘’	Hanelt et al. 2015
KM382303	‘’	Hanelt et al. 2015
KM382302	‘’	Hanelt et al. 2015
KM382301	‘’	Hanelt et al. 2015
KM382300	‘’	Hanelt et al. 2015
KM382299	‘’	Hanelt et al. 2015
KM382297	G. cf. robustus (Clade 5)	Hanelt et al. 2015
KM382296	‘’	Hanelt et al. 2015
KM382295	‘’	Hanelt et al. 2015
KM382294	G. cf. robustus (Clade 4)	Hanelt et al. 2015
KM382293	‘’	Hanelt et al. 2015
KM382292	‘’	Hanelt et al. 2015
KM382291	‘’	Hanelt et al. 2015
KM382290	‘’	Hanelt et al. 2015
KM382289	G. cf. robustus (Clade 3)	Hanelt et al. 2015
KM382288	‘’	Hanelt et al. 2015
KM382287	‘’	Hanelt et al. 2015
KM382286	‘’	Hanelt et al. 2015
KM382285	‘’	Hanelt et al. 2015
KM382284	‘’	Hanelt et al. 2015
KM382283	G. cf. robustus (Clade 2)	Hanelt et al. 2015
KM382282	‘’	Hanelt et al. 2015
KM382281	G. cf. robustus (Clade 1)	Hanelt et al. 2015
KM382280	‘’	Hanelt et al. 2015
KM382279	‘’	Hanelt et al. 2015
KM382278	‘’	Hanelt et al. 2015
KM382277	‘’	Hanelt et al. 2015
KM382318	*G. attoni*	Hanelt et al. 2015
KM382319	‘’	Hanelt et al. 2015
KM382320	*G. balticus*	Hanelt et al. 2015
KM382321	*Gordius* sp. N178	Hanelt et al. 2015
KM382322	*Gordius* sp. N183	Hanelt et al. 2015
KM382323	*Gordius* sp. N297B	Hanelt et al. 2015
KM382324	*Gordius* sp. N357	Hanelt et al. 2015
AB647235	*Gordius* sp. KW-2011-A	Sato et al. 2012
AB647237	*Gordius* sp. KW-2011-B	Sato et al. 2012
AB647241	*Gordius* sp. KW-2011-D	Sato et al. 2012
KY172751	*Gordius* sp. Tobias et al. 2017	Tobias et al. 2017
KY172750	‘’	Tobias et al. 2017
KY172752	‘’	Tobias et al. 2017
KY172759	‘’	Tobias et al. 2017
KY172765	‘’	Tobias et al. 2017
KY172770*	‘’	Tobias et al. 2017
KY172777	‘’	Tobias et al. 2017
KY172749	‘’	Tobias et al. 2017
KY172792	‘’	Tobias et al. 2017
KY172789	‘’	Tobias et al. 2017
KY172791	‘’	Tobias et al. 2017
KY172799	‘’	Tobias et al. 2017
KY172801	‘’	Tobias et al. 2017
KY172802	‘’	Tobias et al. 2017
KY172804	‘’	Tobias et al. 2017
KY172753	*G. paranensis* (Clade2)	Tobias et al. 2017
KY172754	‘’	Tobias et al. 2017
KY172755	‘’	Tobias et al. 2017
KY172756	‘’	Tobias et al. 2017
KY172776	‘’	Tobias et al. 2017
KY172782	‘’	Tobias et al. 2017
KY172813	‘’	Tobias et al. 2017
KY172811	*G. paranensis* (Clade1)	Tobias et al. 2017
KY172812	‘’	Tobias et al. 2017
KX591948	*Acutogordius taiwanensis*	Chiu et al. 2017
KX591947	‘’	Chiu et al. 2017
KX591946	‘’	Chiu et al. 2017
KX591945	‘’	Chiu et al. 2017
KX591944	‘’	Chiu et al. 2017
KX591943	‘’	Chiu et al. 2017
KX591942	‘’	Chiu et al. 2017
KX591941	‘’	Chiu et al. 2017
KX591940	‘’	Chiu et al. 2017
KX591939	‘’	Chiu et al. 2017
KX591938	‘’	Chiu et al. 2017
KX591937	‘’	Chiu et al. 2017
KX591936	‘’	Chiu et al. 2017
KX591935	‘’	Chiu et al. 2017
KX591934	‘’	Chiu et al. 2017
KX591933	‘’	Chiu et al. 2017
KX591932	‘’	Chiu et al. 2017
KX591931	‘’	Chiu et al. 2017
KX591930	‘’	Chiu et al. 2017
KX591929	‘’	Chiu et al. 2017
KX591928	‘’	Chiu et al. 2017
KX591927	‘’	Chiu et al. 2017
KX591926	‘’	Chiu et al. 2017
KX591925	‘’	Chiu et al. 2017
KX591924	‘’	Chiu et al. 2017
KX591923	‘’	Chiu et al. 2017
KX591922	‘’	Chiu et al. 2017
MF983649	Myanmar nematomorph	
Out group		
HM044105	*Chordodes formosanus*	Chiu et al. 2011
HM044124	‘’	Chiu et al. 2011
KY172780	*Euchordodes nigromaculatus*	Tobias et al. 2017
KY172803	‘’	Tobias et al. 2017
KY172747	*Parachordodes diblastus*	Tobias et al. 2017
KY172778	‘’	Tobias et al. 2017

* KY172770 was excluded from the analysis since its high difference from the member of *Gordius* and the high similarity with *Euchordodes nigromaculatus*.

#### Phylogeny.

**Figure 7.**
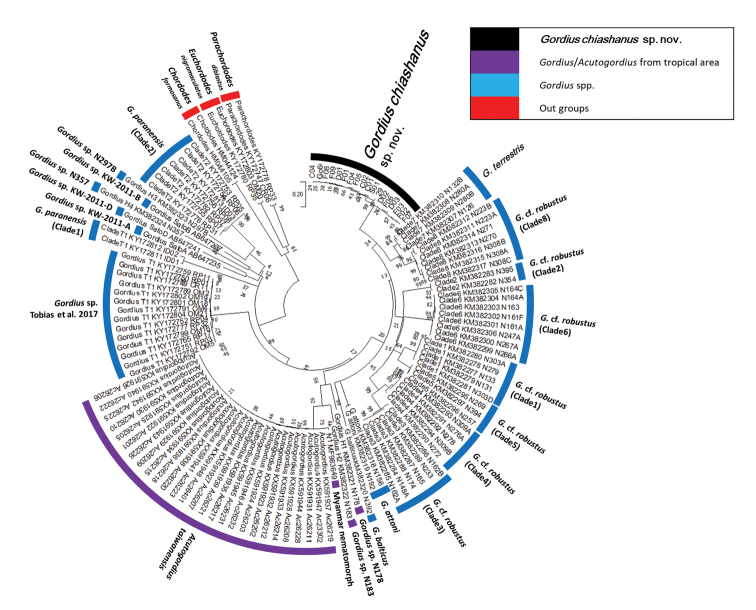
Phylogenetic relationship of *Gordius*/*Acutogordius* spp. restructured using COI partial sequences compared with *C. formosanus*, *E. nigromaculatus*, and *P. diblastus* as out groups. Numbers at the nodes represent the percentage of 1000 bootstrap replicates.

The partial COI sequences of the 18 free-living adults contained 15 haplotypes with 392 invariable sites, nine singletons, and 21 parsimoniously informative sites. The genetic distance among them was 0.0024 within the range of 0.0000–0.0510. The three living adults and six worms inside the hosts were considered conspecific with the 18 free-living adults because of their small genetic distances (0.0000–0.0719). The mean interspecific genetic distances between *Gordius chiashanus* sp. nov. and other *Gordius* species or clades were in the range of 0.2320–0.4242, and that between *Gordius chiashanus* sp. nov. and *Acutogordius taiwanensis* was 0.3648 (Table 3). In addition to short genetic distances, the conspecific status of the 18 free-living adults was also supported because all the samples were located in a single clade, as indicated by a high bootstrap value. No subgroup was detected because the polytomic topology exhibited low bootstrap values and short genetic distances. The *Gordius* species/clades in the present result were consistent with the results of Hanelt et al. (2015) and Tobias et al. (2017), despite slight differences in the relative relationships among species, which might be attributable to the differences in models used or the shorter sequence adopted in previous studies. The clade of *A. taiwanensis* was located within that of the *Gordius* species, and it did not behave as a sister group (Fig. 7).

#### Reproductive season.

**Figure 8.**
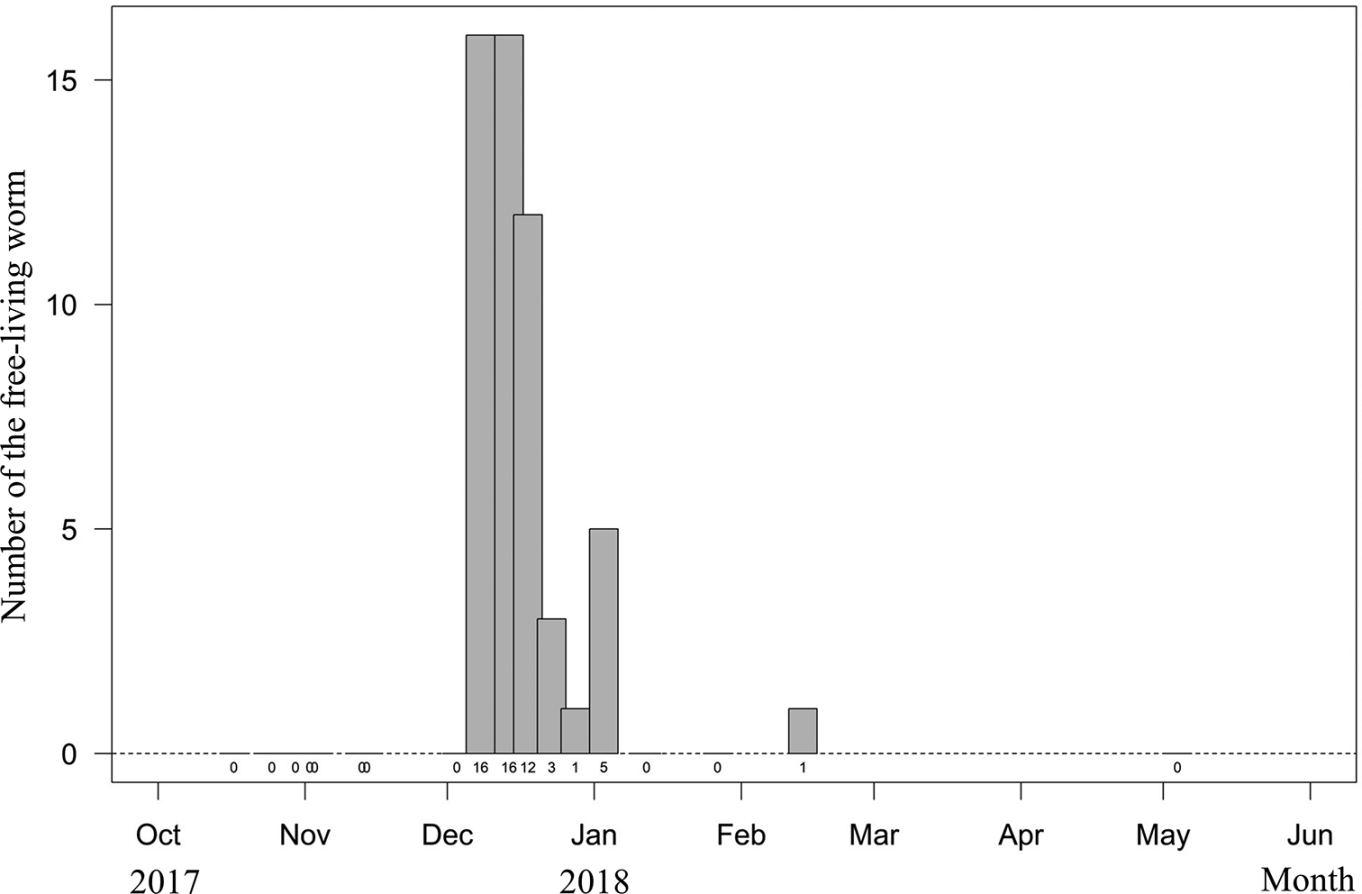
Seasonal occurrence of free-living adults of *Gordius chiashanus* sp. nov. Numbers at the bottom indicate the actual number of each bar.

Free-living adult worms frequently aggregate and mate on wet ground (Fig. 5B, C) after rain or fog, and they are sometimes found in water or soil (Fig. 5D). They suddenly emerge in early December, and their number decreases within 1–2 months (Fig. 8). During the reproductive season, no infected host was found. The seasonality and pattern of *Gordius chiashanus* sp. nov. differed from the graph constructed using data from *C. formosanus* (Chiu et al. 2016).

#### Diagnosis and comments.

The 21 free-living *Gordius* adults and six juvenile worms from round-backed millipedes were judged as belonging to the same species in accordance with the results that they all were located in the same clade in the phylogenetic tree and had low genetic distances (Fig. 7, Table 3). These samples were regarded as a new species, *Gordius chiashanus* sp. nov., on the basis of their distribution patterns of bristles on the male tail and presence of a vertical white stripe on the anterior ventral side and areoles on the inside wall of the cloacal opening.

**Table 3.** Intra- and interspecific mean COI genetic distances of *Gordius*/*Acutogordius* species or clades under K2P model.

Species/Clade		1	2	3	4	5	6	7	8	9	10	11	12	13	14	15	16	17	18	19	20	21	22
1	*Gordius chiashanus* sp. nov.	**0.024**																					
2	G. cf. robustus (Clade1)	0.285	**0.009**																				
3	G. cf. robustus (Clade2)	0.312	0.217	**0.015**																			
4	G. cf. robustus (Clade3)	0.293	0.297	0.275	**0.007**																		
5	G. cf. robustus (Clade4)	0.308	0.208	0.249	0.157	**0.012**																	
6	G. cf. robustus (Clade5)	0.272	0.165	0.211	0.227	0.222	**0.003**																
7	G. cf. robustus (Clade6)	0.293	0.257	0.251	0.255	0.228	0.259	**0.006**															
8	*G. terrestris*	0.232	0.209	0.265	0.250	0.230	0.222	0.238	**0.020**														
9	G. cf. robustus (Clade8)	0.265	0.203	0.307	0.338	0.253	0.244	0.251	0.122	**0.026**													
10	*G. attoni*	0.277	0.229	0.288	0.337	0.274	0.238	0.289	0.231	0.249	**0.010**												
11	*G. balticus*	0.316	0.260	0.298	0.288	0.269	0.274	0.304	0.264	0.323	0.337	–											
12	*Gordius* sp. N178	0.352	0.260	0.313	0.370	0.289	0.340	0.330	0.256	0.290	0.271	0.323	–										
13	*Gordius* sp. N183	0.329	0.302	0.290	0.373	0.317	0.344	0.365	0.294	0.336	0.277	0.301	0.246	–									
14	*Gordius* sp. N297B	0.424	0.416	0.462	0.547	0.441	0.443	0.478	0.375	0.412	0.348	0.455	0.343	0.414	–								
15	*Gordius* sp. N357	0.332	0.366	0.387	0.420	0.376	0.396	0.302	0.357	0.359	0.379	0.439	0.375	0.434	0.441	–							
16	*Gordius* sp. KW-2011-A	0.384	0.325	0.327	0.453	0.371	0.336	0.345	0.347	0.348	0.331	0.376	0.332	0.376	0.372	0.424	–						
17	*Gordius* sp. KW-2011-B	0.334	0.375	0.365	0.370	0.334	0.407	0.364	0.302	0.363	0.333	0.380	0.323	0.358	0.333	0.308	0.290	–					
18	*Gordius* sp. KW-2011-D	0.375	0.300	0.344	0.393	0.373	0.294	0.388	0.388	0.367	0.369	0.405	0.384	0.390	0.374	0.403	0.312	0.301	–				
19	*G. paranensis* (Clade1)	0.369	0.405	0.381	0.450	0.381	0.410	0.359	0.373	0.395	0.409	0.398	0.408	0.466	0.426	0.453	0.415	0.386	0.440	**0.049**			
20	*G. paranensis* (Clade2)	0.337	0.348	0.391	0.436	0.384	0.372	0.368	0.333	0.368	0.345	0.339	0.334	0.385	0.324	0.404	0.357	0.327	0.344	0.377	**0.010**		
21	*Gordius* sp. Tobias et al. 2017	0.335	0.283	0.293	0.436	0.355	0.311	0.366	0.287	0.337	0.347	0.358	0.254	0.308	0.343	0.353	0.304	0.335	0.321	0.354	0.337	**0.012**	
22	*Acutogordius taiwanensis*	0.365	0.343	0.327	0.401	0.386	0.368	0.345	0.322	0.375	0.304	0.336	0.270	0.210	0.462	0.469	0.432	0.376	0.366	0.435	0.389	0.311	**0.002**

-Indicates a single haplotype whose intraspecific distance could not be calculated.

The concentration of bristles and spines on the male tail lobes has been previously described in species from the Palaearctic (Spiridonov 1984; Schmidt-Rhaesa 2010) and Nearctic realms (Anaya et al. 2019). In *Gordius chiashanus* sp. nov., this dense patch of bristles is a stable characteristic that was detected in all samples. The distribution pattern was similar to that of *G. helveticus* (Schmidt-Rhaesa 2010) because the bristles exhibited a progressively broader distribution instead of being concentrated along the row of the ventral border, such as in *G. karwendeli* Schmidt-Rhaesa, 2010 (Schmidt-Rhaesa 2010) and *G. terrestris* (Anaya et al. 2019), or in a circular patch of concentrated spines, such as in *G. spiridonovi* Schmidt-Rhaesa, 2010 (Spiridonov 1984).

Although the distribution pattern of the bristles is similar to that of *G. helveticus*, *G chiashanus* sp. nov. is morphologically distinct because of the presence of stout bristles on the mid-body, a vertical white stripe on the anterior ventral side, and areoles on the inside wall of the cloacal opening. The vertical white stripe on the anterior ventral side can be easily observed by the naked eye, but it has rarely been mentioned thus far. The presence of a white stripe was previously reported in the terrestrial hairworm, *G. terrestris* (Anaya et al. 2019), which exhibits a broad white patch; however, the patch is likely to be the intensive aggregation of white spots in *Gordius chiashanus* sp. nov. The presence of areoles on the inside wall of the cloacal opening has only been reported in an unknown *Gordius* (Schmidt-Rhaesa 2012, fig. 3.2.2). Although cloacal openings are usually covered by contamination in many *Gordius* species, as was the case in most of our samples, the areole on the inside wall of cloacal opening might not be a general characteristic of the genus *Gordius* because it is absent in at least some species (e.g., *G. serratus* Schmidt-Rhaesa, 2010, *G. terrestris*, *G. spiridonovi*) (Schmidt-Rhaesa 2010; Schmidt-Rhaesa and Prous 2010; Anaya et al. 2019).

## Discussion

*Gordius chiashanus* sp. nov. and the two previously described species, namely *A. taiwanensis*Chiu et al., 2017 and *C. formosanus* Chiu, 2011 (Chiu et al. 2011, 2017), are the three most frequently sighted horsehair worm species in Taiwan. Unlike the two low-altitude species, *Gordius chiashanus* sp. nov. inhabits medium altitude areas (1100–1700 m), which matches the distribution of its millipede host, *Spirobolus* sp. nov. (Hsu and Chang, unpublished), in Taiwan (1100–1600 m) (Hsu 2008).

**Morphology of *Gordius chiashanus* sp. nov.** With approximately 90 valid species, *Gordius* is the second most diverse genus of the phylum Nematomorpha (Schmidt-Rhaesa 2012). However, because of the lack of reliable diagnostic characteristics and non-hereditary morphological variation associated with methods of examination, environmental damage, mucus-like structure covering the surface, and different hosts, species identification within this genus is difficult (Schmidt-Rhaesa 2001, 2010; Chiu et al. 2011, 2017; Hanelt et al. 2015). Previously, the white spot has been only found on the male cuticle (Schmidt-Rhaesa 2010). However, we found it, but unexpectedly, in the female *Gordius chiashanus* sp. nov. by examination with a compound microscope. It is clearly necessary to reexamine other species since it might have been ignored especially in the female samples. The mucus-like structure is the structure covering the body surface which might also cause morphological variation. It was first reported in *A. taiwanensis* (Chiu et al. 2017) but not in our observations of *C. formosanus* (Chiu et al. 2011, 2017). In *Gordius chiashanus* sp. nov., it was more obvious than that of *A. taiwanensis* by the bright light reflection on the body surface and the hazy appearance surrounding the worms after treatment with hot water. The mucus-like structure appeared opaque under the SEM; this opacity might hamper the visibility of small structures (Fig. 1C, D), consequently, the reliability of such a diagnostic characteristic is low.

**Adult and larval size.** The body length of *Gordius* is variable and can be longer than 2 m (Schmidt-Rhaesa 2010). Relative to phylogeny, host size and intensity of infection play more crucial roles in determining worm size (Hanelt 2009; Chiu et al. 2017). Although the adult length is less likely to be a common feature shared among a species, larval size might have been overlooked. Hidden diversity due to large cysts in the paratenic host is often detected (Chiu et al. 2016). Larvae of *Gordius chiashanus* sp. nov. are morphologically similar to *A. taiwanensis* (Chiu et al. 2017) but significantly longer than *A. taiwanensis* larvae (preseptum + postseptum: 162.80 ± 1.78 µm vs. 112.00 ± 5.52 µm, larvae treated with hot water). In terms of comparison with other *Gordius* species, although the measurements varied considerably among the untreated larvae, the larval lengths of *Gordius chiashanus* sp. nov. (115.38 ± 12.08 µm) were similar to those of G. cf. robustus # 1 (110.0 µm in Szmygiel et al. 2014) but longer than the unfolded larva of a *Gordius* species (80.02 µm in Fig. 1D, Harkins et al. 2016) and shorter than those of G. cf. robustus # 2 (140.2 µm in Szmygiel et al. 2014). The fine structures of larvae are potential to be adopted in distinguishing the close species. By examining with SEM, Anaya et al. (2019) found differences in the number of spines on the proboscis, while *G. terrestris* has seven spines on the distal end of the left lateral and right lateral sides, whereas there are nine in G. cf. robustus #1 (Szmygiel et al. 2014). Similarly, the pattern of spines on the proboscis is also different in *C. formosanus* (nine on the distal end of the dorsal and ventral sides (Chiu et al. 2011)) and *C. morgani*, *C. kenyaensis*, and *C. janovyi* (5 on the each side) (Bolek et al. 2010, 2013; Szmygiel et al. 2014). In this study, larvae of *Gordius chiashanus* sp. nov. are failed to be examined by SEM, but it is worth to compare the larval morphology through the horsehair worm species in future studies.

**Phylogenetic relationship of *Gordius* and *Acutogordius*.** Molecular comparisons have been rarely conducted in the 19 nematomorph genera (Bleidorn et al. 2002; Efeykin et al. 2016), and the present study is the first examination of the phylogenetic relationship of *Acutogordius* and *Gordius* belonging to the family Gordiidae. Because of the shared characteristic of the postcloacal crescent, *Acutogordius* was considered to be phylogenetically close to *Gordius* but distinct because of its pointed tail lobes (Schmidt-Rhaesa 2002). Two hypotheses have suggested that *Acutogordius* might act as a sister group or a subtaxon of *Gordius* (Schmidt-Rhaesa 2002). Our results indicate that the genus *Acutogordius* is a subtaxon of *Gordius* species, although including only one *Acutogordius* species in analysis is insufficient to support a monophyly of the genera *Gordius* and *Acutogordius*. Moreover, our results suggest that *Acutogordius* might be a group of *Gordius* that adapts to tropical habitats. The three clades of tropical horsehair worms are grouped together with the sequences for *A. taiwanensis* from Taiwan, one sequence from Myanmar (Myanmar nematomorph, MF983649), *Gordius* sp. N178 (KM382321) from Nicaragua, and *Gordius* sp. N178 (KM382322) from Malaysia. The adaptation to the tropical habitat of these two genera corresponds with the global distribution. *Acutogordius* species are mostly distributed in the lower latitude regions; by contrast, the *Gordius* species mainly inhabits the Palaearctic realm (Schmidt-Rhaesa 2002, 2014; Schmidt-Rhaesa and Geraci 2006; Schmidt-Rhaesa and Schwarz 2016; Chiu et al. 2017). In addition, similar patterns were observed in the altitudinal distribution of these two genera in Taiwan. *Acutogordius taiwanensis* mainly inhabits low-altitude rivers (Chiu et al. 2017), whereas *Gordius chiashanus* sp. nov. is only found in mountains at 1000 m. It is worth to note that *Gordius chiashanus* sp. nov. is in the same clade with *G. terrestris* and G. cf. robustus (clade 8). Despite not highly supported by the bootstrap method, these three species show a distinct similarity in biology. The definitive host of G. cf. robustus (clade 8) is the millipede, whereas that of most of G. cf. robustus (clade 2, 3, 4, 6) are orthopterans (Hanelt et al. 2015). For *Gordius chiashanus* sp. nov. and *G. terrestris*, the egg with a distinct membrane around the larva and the free-living adapting to terrestrial environment have never mentioned in other species. This clade of *Gordius* might represent a unique life history of the horsehair worm.

**Definitive host and route of transmission.** The millipede has been known to be the host of horsehair worms, including the genera *Gordius* and *Gordionus* (Schmidt-Rhaesa et al. 2009; Schmidt-Rhaesa 2012; Hanelt et al. 2015). As a detritivore, it is less likely to ingest horsehair worm cysts from the paratenic host. In 1930, Dorier suggested water and vegetation possible route of transmission after observing the formation of horsehair worm cysts in the external environment instead of inside the paratenic host (reviewed in Schmidt-Rhaesa et al. 2009). Recent observations of free-living cysts support this hypothesis (Bolek et al. 2015; Chiu et al. 2017). However, a detritivore definitive host can also be infected by ingesting corpses of the infected paratenic hosts. The cysts, which were putatively identified as *Gordius chiashanus* sp. nov., found in the mayfly naiads suggest that this is a possible route of transmission. However, the prevalence was low (3.85 and 8.33% from 26 and 24 hosts collected in Shihjhuo in the end of July). It might suggest the less efficiency in transmission or the under estimation of the prevalence since the samples were collected 4 months before the worm appeared on the soil surface.

**Host and host manipulation of horsehair worms.** The host and biological characteristics of *Gordius chiashanus* sp. nov. suggest an atypical life history. In general, freshwater horsehair worms (gordiids) develop in terrestrial definitive hosts and reproduce in aquatic environments (Hanelt et al. 2005). Adult worms maturing in terrestrial hosts have long been observed and confirmed through experimentation to manipulate host behavior to facilitate host falling into water, which enables them to reproduce in water (Thomas et al. 2002; Sanchez et al. 2008; Ponton et al. 2011). However, these observations are confined to the gordiids parasitizing a few host taxa (mantids and orthopterans) (Schmidt-Rhaesa and Ehrmann 2001; Thomas et al. 2002), whereas that parasitizing other hosts, crossing several arthropod taxa (Schmidt-Rhaesa 2010; Bolek et al. 2015), is likely to exhibit the different reproductive strategy. The alternative nonmanipulative hypotheses include the “chance hypothesis” suggested by observations of adult *C. ferganensis* Kirjanova & Spiridonov, 1989 emerging from mantids that drowned in small puddles formed by heavy rains (Kirjanova and Spiridonov (1989), reviewed by Schmidt-Rhaesa and Ehrmann (2001)). The “aquatic life cycle hypothesis” is suggested by the *Gordius* spp. parasitizing aquatic caddisfly larvae as definitive hosts (Valvassori et al. 1988; Schmidt-Rhaesa and Kristensen 2006), and the “terrestrial life cycle hypothesis” suggested by *G. terrestris* laying eggs in wet soil (Anaya et al. 2019).

In this study, the female adult oviposited in the water. The cysts found in the aquatic paratenic hosts and the eggs developing in water also suggest the life cycle of *Gordius chiashanus* sp. nov. could occur in water and on land. However, the current evidence did not exclude the oviposition in the terrestrial environment because no terrestrial paratenic host was examined for cysts. In addition, the double membraned egg (Anaya et al. 2019) and the mating on the ground both suggest *Gordius chiashanus* sp. nov. might be able to reproduce in the terrestrial environment. Regardless of the scenarios, the adult worm might not be carried to water by manipulating behavior of its millipede host. Alternatively, they may emerge in the terrestrial environment, and move into the water or reproduce in the soil. Free-living adults of *Gordius chiashanus* sp. nov. are frequently found moving and mating on the surface of wet soil during periods of fog and rain. The mucus-like structure, which causes a rainbow-like reflection, might endow the worm with a high tolerance to dehydration. In the winter (late November to early February), the number of free-living adults sampled from the surface of the soil, suddenly increased and then steadily diminished. The adult *C. formosanus* has a pattern that differs from the bell curve in terms of its presence inside a manipulated host (Chiu et al. 2016, fig. 8) and free-living adults of *G. difficilis* in the water (Bolek and Coggins 2002). This difference suggests that the seasonal occurrence of *Gordius chiashanus* sp. nov. does not represent the time when the worm matures but the time of reproduction after the free-living adult has waited for suitable soil conditions. That worms emerging from the hosts in the soil might explain why infected millipedes are rarely found on the ground.

## Supplementary Material

XML Treatment for Gordius chiashanus

## Supplementary materials

Supplementary material 1 Video S1 Data type multimedia Explanation note Free-living adult of *Gordius chiashanus* sp. nov. moving on the ground at night, with a rainbow-like reflection on the body surface. https://binary.pensoft.net/file/421536This dataset is made available under the Open Database License (http://opendatacommons.org/licenses/odbl/1.0/). The Open Database License (ODbL) is a license agreement intended to allow users to freely share, modify, and use this Dataset while maintaining this same freedom for others, provided that the original source and author(s) are credited.Ming-Chung Chiu, Chin-Gi Huang, Wen-Jer Wu, Zhao-Hui Lin, Hsuan-Wien Chen, Shiuh-Feng Shiao

Supplementary material 2 Fasta 2 Data type molecular data Explanation note COI sequences adopted in the phylogenetic analysis, including for *Gordius*/*Acutogordius* spp. with *Chordodes formosanus*, *Euchordodes nigromaculatus*, and *Parachordodes diblastus* as out groups. https://binary.pensoft.net/file/421537This dataset is made available under the Open Database License (http://opendatacommons.org/licenses/odbl/1.0/). The Open Database License (ODbL) is a license agreement intended to allow users to freely share, modify, and use this Dataset while maintaining this same freedom for others, provided that the original source and author(s) are credited.Ming-Chung Chiu, Chin-Gi Huang, Wen-Jer Wu, Zhao-Hui Lin, Hsuan-Wien Chen, Shiuh-Feng Shiao
